# Timely Initiation of Antenatal Care and Associated Factors among Pregnant Women Attending at Wachemo University Nigist Eleni Mohammed Memorial Comprehensive Specialized Hospital, Hossana, Ethiopia: A Cross-Sectional Study

**DOI:** 10.1155/2023/7054381

**Published:** 2023-03-16

**Authors:** Dagmawit Tessema, Amanuel Kassu, Amanuel Teshome, Ritbano Abdo

**Affiliations:** ^1^Department of Midwifery, Leku General Hospital, Sidama, Ethiopia; ^2^Department of Midwifery, College of Medicine and Health Science, Wachemo University, Hossana, Ethiopia

## Abstract

**Background:**

Timely detection and treatment of pregnancy-related or preexisting diseases, health education, and the promotion of adequate care provision improve the health of mothers and unborn children. As such, these factors are crucial within the first pregnancy trimester. However, very few women in low and middle-income countries initiate their first ANC in the recommended trimester of pregnancy. This study is aimed at assessing the prevalence of timely initiation of ANC and its associated factors among pregnant women attending antenatal clinics in Wachemo University Nigist Eleni Mohammed Memorial comprehensive specialized hospital, Hossana, Ethiopia.

**Methods:**

A hospital-based cross-sectional study was conducted from April 4, 2022 to May 19, 2022. A systematic sampling technique was used to select study participants. Data were collected from pregnant women using a pretested structured interview questionnaire. EpiData version 3.1 was used to enter the data, and SPSS version 24 was used to analyze it. Bivariate and multivariable logistic regression were used to identify the associated factors at a 95% confidence interval with a *p* value < 0.05.

**Results:**

This study indicated that 118 (34.3%) of the women initiated ANC timely. The factors associated with timely initiation of ANC included women aged 25–34 years (AOR = 0.3; 95% CI: (0.1, 0.7)), tertiary maternal education (AOR = 3.2, 95% CI: (1.0, 9.9)), zero parity (AOR = 7.7; 95% CI: (3.6, 15.3)), planned pregnancy (AOR = 13.7; 95% CI: (5.5, 34.3)), good knowledge about ANC services (AOR = 3.1; 95% (CI: (2.3, 11.3)), and good knowledge about danger signs in pregnancy (AOR = 4.8; 95% CI: (2.2, 8.1)).

**Conclusion:**

This study demonstrates the importance of making a significant effort to increase the coverage of timely ANC initiation in the study area. Therefore, increasing the awareness level of mothers regarding ANC services given during pregnancy and danger signs in pregnancy and advancing the academic level of mothers are essential to increase the coverage of timely initiation of ANC.

## 1. Introduction

Antenatal care (ANC) is the care provided by skilled healthcare professionals to women throughout their pregnancy. It is essential to protect the health of women and their unborn children [1, 2]. In 2016, the World Health Organisation (WHO) developed a new ANC model to improve the quality of antenatal care. It recommends that every pregnant woman should start her first ANC session before the gestational age of 12 weeks [2]. A timely initiation of ANC increases the number of maternal and fetal assessments. These assessments can detect complications and improve communication between health providers and pregnant women, increasing the likelihood of positive pregnancy outcomes [2]. Beginning ANC within the first trimester facilitates the adoption of preventive measures, the early detection of diseases, and the provision of relevant and up-to-date information. It can also encourage the integration of clinical practices, the provision of psychosocial and emotional support, the reduction of pregnancy-related complications, and the elimination of health inequalities [2–5]. Crucially, it helps to reduce maternal and neonatal morbidity and mortality rates [4, 5]. Initiating ANC visits during the first trimester provides the best opportunity for conveying the key components of maternity healthcare services and increasing retention within the maternity care pathway [6–10].

Globally, the ANC initiation rate is 58.6% but varies according to the continent. The estimated rate of early antenatal care visits is 48.1.0% in low-income countries compared with 84.8% in high-income countries [11]. In sub-Saharan Africa (SSA), the initiation of ANC visits within the first trimester is 38.0%. This statistic ranges from 14.5% in Mozambique to 68.6% in Liberia [12]. According to the Mini Ethiopian demographic and health survey (EDHS) reports from 2019, only 28% of women had their first ANC visit during the first trimester. This rate also varies across geographic regions [13].

Existing evidence indicated that the prevalence and associated factors varied between regions and within regions [14–25]. Furthermore, variations in the definition of timely ANC initiation were identified [23, 26–29]. Finding the single figure and identifying associated factors in the study area is essential to designing appropriate interventions. This study is aimed at assessing the prevalence of timely ANC initiation and its associated factors among pregnant women attending antenatal clinics at Wachemo University Nigist Eleni Mohammed Memorial, a comprehensive and specialized hospital, in Hossana, Ethiopia.

## 2. Materials and Methods

### 2.1. Study Design, Period, and Setting

A hospital-based cross-sectional study was conducted at Wachemo University Nigist Eleni Mohammed Memorial comprehensive specialized hospital from April 4, 2022 to May 19, 2022. Nigist Eleni Mohammed Memorial Comprehensive Hospital is one of the teaching and referral hospitals in southern Ethiopia. The hospital is located in Hadiya zone, Hosanna town, Ethiopia, at a distance of 232 km south of Addis Ababa. The hospital serves about 3.2 million people per year and provides services such as outpatient treatment, emergency treatment, surgery, delivery, laboratory, pharmacy, and mental health care. The hospital has medical, obstetrics and gynaecology, surgical, orthopaedic, and paediatric wards and approximately 350 beds with a total of 720 health workers. In the maternal and child health care unit, the number of mothers who attended antenatal care during the study period was around 726.

### 2.2. Source Population and Study Population

Pregnant women who attended an antenatal care clinic in a hospital during the study period and the study population comprised of randomly sampled pregnant women from this group. Pregnant women with mental illness and those who were unable to hear and talk were excluded from the study because it was considered that they would not be able to provide the necessary information.

### 2.3. Sample Size and Sampling Procedure

The single population proportion formula was used to determine the sample size with the following assumptions: the proportion of timely initiation of ANC was taken from the study conducted in the Kambata Tembaro zone (33.8%) [29], with a 95% confidence interval (CI), a margin error of 5, and a 5% nonresponse rate; the final sample was 348 pregnant mothers. A systematic sampling technique was applied to select 361 study participants. First, the interval was estimated by dividing the total number of pregnant women who attended ANC during the data collection period (*n* = 726/*n* = 361), which was estimated from the previous six-month client follow up. Finally, we chose the first pregnant woman randomly from the first two women, and then every two pregnant women were included. With 17 pregnant women declining to participate in the study, we had an overall response rate of 95.3%.

### 2.4. Data Collection Tool and Quality Control

A structured face-to-face exit interviewer-administered questionnaire was used to collect data. The questionnaire was developed based on previous studies [14–35]. The tool was initially prepared in English, first translated into the local language, and then translated back into English by experts to check consistency. Data were collected by three midwives who interviewed the mothers after explaining the details of the study. The questionnaire was pretested on a sample of 18 pregnant women at Bobicho health centre, and some modifications were made after their feedback. The data collectors and supervisors were trained for a day by the investigators of this study on the content of the questionnaire and the manner of data collection. The completeness and consistency of the variables during data entry and analysis were confirmed using frequency distributions.

### 2.5. Variables

#### 2.5.1. Dependent Variable

Timely initiation of ANC

#### 2.5.2. Independent Variables

Religion (Orthodox, Protestant, Muslim, and Catholic); marital status (married or single); women's education (primary and below, secondary level, or tertiary education); women's age in years (15-25, 25-34, or ≥35 years); husband's education (primary and below, secondary level, or tertiary education); and husband's occupation (merchant, government employee, or NGO/private) and average monthly income (below or above median). Obstetric factors included the following: gravidity (primigravida, multigravida, or grand multigravida); number of parity (zero or “one or more”); previous pregnancy ANC follow up (yes or no); time of ANC initiation in previous pregnancy (first, second or third trimester); abortion history (yes or no based on women report); current ANC follow up (yes or no based on mother's self-report); frequency of ANC (two, three, or four), knowledge regarding ANC service (poor, moderate, or good), knowledge regarded ANC survives (poor, moderate, or good), pregnancy status (planned or unplanned) decision made to ANC services (women, husband, or both), and pregnancy-related problems (yes or no).

### 2.6. Operational Definition

*Timely initiation of ANC:* this means that the first antenatal contact was before 12 weeks (during first trimester) [2].

*Knowledge regarding ANC service:* we measured using five items with “yes” or “no” responses. Participants' responses were summed, and the knowledge scores were calculated for each participant. Finally, by using modified Bloom's cut-off point, categorization was made as, 80%-100% good knowledge, 50-79% moderate, and <50% poor knowledge.

*Knowledge regarding ANC service:* we assessed using seven items, with a correct answer being given a score of “1” and an incorrect answer being given a score of “0.” Participants' overall knowledge was categorized, using modified Bloom's cut-off point, as good if the score was between 80% and 100%, moderate if the score was between 50% and 79%, and poor if the score was less than 50% [36, 37]. Average monthly income is categorized based on median monthly income, above or below the median.

*Primigravida:* a woman who has been pregnant once or is currently pregnant for the first time.

*Multigravida:* a woman who has been pregnant two or more times.

### 2.7. Data Analysis

Epi-Data version 3.1 was used to enter data, which was then exported to the Statistical Package for Social Science (SPSS) version 24.0 software for analysis. Frequency and proportion were used to summarize and present major findings. Initially, bivariate logistic regression was performed for the selection of candidate variables for the multivariable logistic regression. In bivariate logistic regression, the variables with a *p* value ≤ 0.25 were entered into the multivariable logistic regression model. It was conducted to determine the independent associated factors of the outcome variable and control possible confounders. An odds ratio was accepted at a 95% CI, and a *p* value < 0.05 was stated as statistically significant. The appropriateness of the model was by the Hosmer-Lemeshow statistic test and area under ROC curve.

### 2.8. Ethical Consideration

Ethical clearance was obtained from research review and ethics committee of College of Medicine and Health Sciences, Wachemo University. Additionally, legal permission was obtained from the hospital. The respondents were informed about the objective and purpose of the study and their right to withdraw at any time. Then, written consent was obtained from each respondent. Informed assent was obtained from a parent or guardian for study participants younger than 18 years of age. Confidentiality was maintained throughout the study by excluding personal identifiers, such as names and addresses.

## 3. Results

### 3.1. Description of Study Participants

Three hundred forty-four (98.8%) pregnant women participated in the study. Nearly three-fourths of the women surveyed 245 (71.2%) were aged 25–34 years, with a sample mean age of 28.0 years. The majority of the mothers, i.e., 253(73.4%) were Hadiya ethnic, 240 (69.8%) were Protestants, and 335(97.4%) and 207(60.27%) were housewives. The results of other sociodemographic factors characteristics are indicated in Table 1.

### 3.2. Obstetric-Related Characteristics and Timely Initiation of ANC

**Figure 1:**
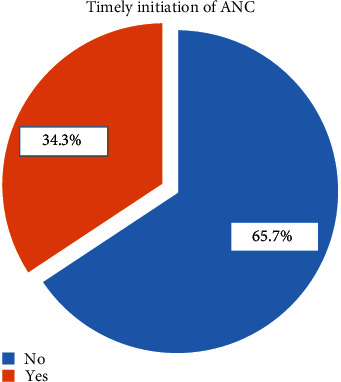
Prevalence of timely initiation of ANC among pregnant women attending ANC clinic in Wachemo University Nigist Eleni Mohammed memorial comprehensive specialized hospital, 2022.

Of the 344 study participants, 111(32.3%) were primigravida, and 158(45.9%) and 75(21.8%) were multigravida and grandmultigravid. Forty-one (11.9%) participants had ever experienced abortion; 8 (2.3%) had a history of at least one child death; and 6 (1.7%) had a history of at least one stillbirth. Thirty-three (9.6%) women had problems during their last delivery. More than half (57.3%) of the respondents have a previous history of attending ANC services, and 21.2% of them started their visit in the first trimester of their last pregnancy. Forty-eight (14.0%) women have faced obstetric complications during the current pregnancy. In terms of participants' knowledge of antenatal care services provided, 114 (33.1%) had a good knowledge level, while 230 (52.6%) had a poor knowledge level. Regarding the participants' knowledge of danger signs in pregnancy, nearly one-fourth (22.1%) of the participants were knowledgeable. In terms of women's autonomy, 210 (61.0%) decided to begin their follow up on their own, 78 (22.7%) by their husbands, and 56 (16.3%) by both (Table 2). This study indicated that 118 (34.3%) of the women initiated ANC timely (Figure 1).

### 3.3. Factors Associated with Timely Initiation of ANC Visit

In bivariate logistic regression analysis, maternal age, women's and husband's level of education, parity, planned pregnancy, knowledge of ANC services, and knowledge of pregnancy danger signs during pregnancy were associated with the timely initiation of an ANC visit at *p* ≤ 0.25. These variables were entered into a multivariable logistic regression to determine their statistical significance in relation to the timely initiation of ANC. Maternal age (25-34 years), women's education level (tertiary education), parity (zero), pregnancy status (planned), knowledge of pregnancy danger signs (good), and knowledge of ANC services (good) were found to be significantly associated with timely initiation of ANC at *p* value < 0.05 after performing multivariable logistic regression analysis.

Women aged 25–34 years (AOR = 0.3; 95% CI: (0.1, 0.7)) were 70% less likely to initiate their first antenatal care visit on time compared to women aged ≤ 25 years. Tertiary maternal education was significantly associated with higher odds of timely initiation of an ANC visit (AOR = 3.2, 95% CI: (1.1, 9.9)). Women with planned pregnancies were more likely to begin ANC visits on time (AOR = 13.7; 95% CI: (5.5, 34.)). Furthermore, women who had zero parity were more likely to have a timely ANC visit (AOR = 7.4; 95% CI: (3.6, 15.3)) than primiparous mothers. Respondents who have good knowledge about ANC services are 3.1 times more likely to initiate an ANC visit late than those who have good knowledge about ANC services (AOR = 3.1; 95% CI: (2.3, 11.3)). Moreover, the likelihood of timely initiation of an ANC visit increased in women whose knowledge score regarding pregnancy danger signs was higher (AOR = 4.8, 95% CI: (2.2, 10.2)) (Table 3).

## 4. Discussion

To ensure that women receive optimal care and complete the recommended number of ANC sessions, the WHO's 2016 ANC model advises scheduling the first ANC appointment during the first trimester of pregnancy. This recommendation focuses on reducing pregnancy-related complications and stillbirths. Attaining sustainable development goal 3 is not possible without ensuring that every woman uses ANC at an optimal rate in accordance with the recommendations. In this study, the prevalence rate of timely initiation of ANC was observed to be 34.3% (95% CI: 29.4–39.2), which was similar to rates reported in studies conducted in Boditi, Southern Ethiopia (38%) [15], Kambata, Southern Ethiopia (31.4%) [29], Afghanistan (33.1%) [22], Cameroon (35.6%) [30], and SSA (38.0%) [12]. By contrast, a lower prevalence of timely initiation of ANC was observed in this study compared to those of studies conducted in Ethiopia in Bahir Dar (44.2%) [17], Goba (61.2%) [16], Gondar (47.5%) [14], and Wollo (40.5%) [31]. The higher proportions of timely ANC initiation could be attributed to increased service awareness.

In this study, the prevalence of timely initiation of ANC was higher than that in studies done in Shebedino District, Sidama Region, Southern Ethiopia (21.7%) [18], and Southwest Ethiopia (28.8%) [34]. This may have been because the previous studies were conducted in a rural district, where women have low awareness levels about maternal healthcare services. This difference could also be explained by regional variation within Ethiopia.

The results of this study also show that the prevalence of timely initiation of ANC services was lower compared to those in other countries, such as Benin (45.6%) [7], Saudi Arabia (75.0%) [21], Ghana (68.0%) [24], and Uganda (50%) [25]. Possible reasons could be variations in socioeconomic status, better access, and higher awareness regarding services. Notably, the timely initiation of ANC visits in this study was much higher than reported in rural districts in Northern Ghana (8.9%) [32] and Kenya (7.3%) [33]. These differences could be explained by variations in the study settings; the study in Ghana covered more rural areas and the sample size in the study conducted in Kenya was smaller.

In this study, women aged 25–34 years were 70% less likely to initiate ANC in a timely manner than women younger than 25 years. This conclusion aligns with the findings of studies carried out in Ethiopia [18, 24, 35, 36], but it is at odds with studies done in Myanmar [37], Ghana, and SSA [12], which found that women under the age of 25 were less likely to commence ANC service on time.

Women's higher education was significantly associated with the timely initiation of ANC services. This conclusion aligns with research from Ethiopia [16, 18, 35], Nepal [23], Myanmar [26], and Ghana [32]. This may be explained by the fact that education can increase women's understanding, access to information, effectiveness in absorbing advocacy messages from the media, and ability to work in the healthcare industry. In addition, women might be better informed about the services offered that make use of maternity and child health care.

Women with zero parity were likelier to start receiving ANC services on time than women with high parity (one or more). This conclusion aligns with research from Ethiopia [18, 31, 38], Saudi Arabia [21], Ghana [24], and Myanmar [26]. This conclusion may be explained by the fact that women who have never been pregnant or birth before are likelier to be anxious about finding out whether they are pregnant and to think that starting ANC as soon as possible is crucial.

Compared to women who had an unplanned pregnancy, those who had a planned pregnancy started their ANC services earlier, as shown in studies conducted in Ethiopia [14, 27, 31, 35, 38], Nepal [23], Uganda [25], Myanmar [26], and Cameroon [30]. This conclusion may be explained by the fact that pregnant women may prioritise maternal health care because they desire a healthy pregnancy and child.

Women who had a high level of knowledge about pregnancy danger signs were more likely to start ANC timely compared with less knowledgeable women. This might be due to the fact that the more mothers know about the danger signs, the more they use them [38–40]. In addition, women who are knowledgeable about danger signs are likelier to work on preventing dangerous situations before they occur.

Regarding the timely initiation of ANC service, knowledgeable women were likelier to initiate ANC service in a timely manner compared with nonknowledgeable women. This finding is again supported by studies conducted in Ethiopia [14, 19, 27, 35, 38]. A possible justification might be that women who have good knowledge of maternal healthcare are directly linked to higher maternal healthcare utilisation because knowledgeable women might have a better understanding of their health, which contributes to their acceptance and utilisation of healthcare services.

## 5. Limitations of the Study

Hospital samples may not be representative of the entire community. In addition, the data were collected using a face-to-face exit interviewer questionnaire; therefore, there could be recalled biases in reporting the time of first ANC contact, especially for pregnant women who have more than one visit. Additionally, this study was not supported by a qualitative method.

## 6. Conclusion

Even if the coverage of timely initiation of ANC in a study area is higher than the national figure, there is still a high percentage of women who do not start their first ANC visit in the first trimester of pregnancy. This study demonstrates the importance of making a significant effort to increase the coverage of timely ANC initiation in the study area. The Zonal Health Department and respective health officials develop specific interventions to increase coverage of timely ANC initiation, with a focus on these underutilized first contacts for ANC. Health professionals and care providers should provide great attention to creating awareness regarding ANC services provided during pregnancy, pregnancy-related danger signs, and women who have low parity. The results of the study will help policy-makers, program designers, and nongovernmental organizations to support the study area. Additionally, the findings of the study can also be used as reference for further study in the same area of inquiry. Finally, we recommend further research to be done at community level and areas supplemented with qualitative data.

## Figures and Tables

**Table 1:** Sociodemographic characteristics of pregnant women attending ANC at Wachemo University Nigist Eleni Mohammed memorial comprehensive specialized hospital Hossana, Ethiopia, 2022.

Variables	Categories	Frequency (*n* = 344)	Percentage (%)
Women age in year	15-25	72	20.9
	25-34	245	71.2
	≥35	27	7.8
Ethnicity	Hadiya	253	73.5
	Kambata	43	12.5
	Silte	23	6.7
	Others^∗^	25	7.3
Religion	Orthodox	47	13.7
	Protestant	240	69.8
	Muslim	44	12.8
	Catholic	13	3.8
Marital status	Married	335	97.4
	Single	9	2.6
Women's education	Primary education and below	189	54.9
	Secondary education	111	32.3
	Tertiary education	44	12.8
Women's occupation	Housewife	207	60.2
	Merchant	47	13.7
	Daily worker	56	16.3
	Government employee	34	9.9
Husband education	Primary and below	75	21.8
	Secondary education	109	31.7
	Higher education	160	46.5
Husband's occupation	Merchant	216	62.8
	Government employee	74	21.5
	Daily worker	19	5.5
	NGO/private	35	10.2
Average monthly income	Below median	234	68.0
	Above median	110	32.0

**Table 2:** Obstetric characteristics of pregnant women attending antenatal care at Wachemo University Nigist Eleni Mohammed memorial comprehensive specialized hospital, Hossana, Ethiopia, 2022.

Variable	Category	Frequency	Percent (%)
Gravidity	Primigravida	111	32.3
	Multigravida	158	45.9
	Grand multigravida	75	21.8
Parity	Zero	117	34.0
	One and more	227	66.0
Abortion	Yes	41	11.9
	No	192	55.8
Pregnancy status	Not planned	93	27.0
	Planned	251	73.0
Previous pregnancy ANC (*n* = 233)	Yes	197	88.3
	No	36	11.7
Time of first ANC in previous pregnancy	First trimester	73	37.0
	Second or third trimester	124	63.0
Frequency of ANC visits in current pregnancy	First time	156	45.3
	Two times	80	23.3
	Three times	66	19.2
	Four or more	42	12.2
Knowledge regarding danger signs in pregnancy	Poor	218	63.4
	Moderate	50	14.2
	Good	76	22.1
Knowledge regarded ANC survives	Poor	230	52.6
	Moderate	49	14.2
	Good	114	33.1
Decision made to initiate ANC	Women	210	61.0
	Husband	78	22.7
Pregnancy-related problems	Both	56	16.3
	Yes	48	14.0
	No	296	86.0

**Table 3:** Factors associated with timely initiation of ANC at Wachemo University Nigist Eleni Mohammed memorial comprehensive specialized hospital, Hossana, Ethiopia, 2022.

Variables	Initiation of ANC		OR at 95% CI			
	Late	Timely	COR	*p* value	AOR	*p* value
*Age group*						
15-25 years (ref.)	46	26	1		1	
25-34 years	63	182	0.2 (0.1, 0.3)	0.00^∗∗^	0.3 (0.1, 0.7)	0.01^∗^
≥35years	9	18	3.5 (1.4, 2.7)	0.01^∗^	0.6 (0.2, 2.5)	0.49
*Women's education*						
Primary and below (ref.)	49	140	1			
Secondary	47	64	2.1 (1.2, 3.4)	0.00^∗∗^	1.7 (0.8, 3.4)	0.17
Tertiary education	22	22	2.8 (1.4, 5.6)	0.00^∗∗^	3.2 (1.1, 9.9)	0.03^∗^
*Women's occupation*						
House wife (ref.)	70	137	1		1	
Merchant	13	34	0.7(0.4, 1.5)	0.21	0.5(0.2, 1.4)	0.5
Daily worker	21	35	1.2 (0.6, 2.2)	0.60	0.4 (0.1, 1.7)	0.6
Government	14	20	1.3 (0.6, 2.9)	0.41	0.9 (0.3, 3.1)	0.9
*Husband's occupation*						
Merchant (ref.)	79	137	1		1	
Gov't employee	27	47	0.9 (0.6, 1.7)	1.0	0.6 (0.2, 1.7)	0.35
Daily worker	5	14	0.6 (.2, 1.7)	0.38	0.4 (0.1, 1.8)	0.23
NGO/private	7	28	0.4 (0.8, 1.1)	0.06	0.1 (0.1, 1.1)	0.06
*Husband's education*						
Primary and below (ref.)	20	55	1		1	
Secondary	39	70	1.5 (0.8, 2.9)	0.20	1.1 (4(0.5, 2.7)	0.76
Higher education	59	101	1.6 (0.9, 2.9.7)	0.12	1.3 (0.5, 3.5)	0.63
*Number of parity*						
Zero	65	52	4.1 (2.5, 6.6)	0.00^∗∗^	7.4 (3.6, 15.3)	0.00^∗∗^
One or more parity (ref.)	53	174	1		1	
*Planned pregnancy*						
No (ref.)	12	81	4.9 (2.6, 9.5)	0.00^∗∗^	13.7 (5.5, 34.2)	0.00^∗∗^
Yes	106	145	1		1	
*Knowledge of ANC service*						
Poor (ref.)	74	134	1		1	
Moderate	52	62	0.3 (0.1, 0.7)	0.00^∗∗^	1.8 (0.7, 3.5)	0.2
Good	19	30	4.7 (2.7, 8.3)	0.00^∗∗^	3.1 (2.3, 11.3)	0.00^∗∗^
*Knowledge regarding pregnancy danger signs*						
Poor (ref.)	63	155	1		1	
Moderate	50	26	1.8 (0.9, 3.5)	0.08	0.2 (0.1, 1.8)	0.22
Good	5	45	2.3 (1.4, 3.9)	0.00^∗∗^	4.8 (2.2, 10.2)	0.00^∗∗^
*Decision made to maternal healthcare services*						
Myself	89	133	2.3 (1.2, 4.3)^∗^	0.01^∗^	2.3 (0.8, 5.2)	0.06
Husband (ref.)	16	55	1		1	
Both	13	38	1.2 (0.5, 2.7)	0.7	0.5 (0.5, 3.51)	0.33

## Data Availability

The datasets used and/or analyzed during the current study are available from the corresponding author on reasonable request.

## Abbreviations

ANC: Antenatal care
AOR: Adjusted odds ratio
CI: Confidence interval
EDHS: Ethiopia demographic and health survey
ICC: Interclass correlation coefficient
LMICs: Low- and middle-income countries
NEMMCSH: Nigist Eleni Mohammed memorial comprehensive specialized
OR: Odds ratios
PNC: Postnatal care
SBA: Skilled birth attendance
SPSS: Statistical package for social sciences
SSA: Sub-Saharan Africa.
